# Heated tobacco products and cigarette marketing in nightclubs in Gdansk, Poland: A mixed-methods analysis

**DOI:** 10.18332/tpc/174573

**Published:** 2024-01-05

**Authors:** Julia Nowicka, Lukasz Balwicki

**Affiliations:** 1 Department of Public Health and Social Medicine, Medical University of Gdansk, Gdansk, Poland

**Keywords:** young adults, tobacco marketing, advertising and promotion, heated tobacco products, nightclubs, Poland

## Abstract

**INTRODUCTION:**

Although advertising and promotion of tobacco products in Poland are prohibited, tobacco companies exploit legal loopholes and insufficient enforcement of the laws to conduct their marketing. To reach young adults, advertisements are placed in entertainment and social venues. This aspect of tobacco product marketing in Poland remains under-researched. The main aim of this study was to investigate the occurrence and characteristics of advertising and promotion of tobacco products in nightclubs in Gdansk, a large city in northern Poland.

**METHODS:**

We conducted a single-center observational study of nightclubs in Gdansk, between July and October 2022. Two independent observers visited 30 nightclubs and collected information using pre-prepared research form. The occurrences of tobacco branded bar accessories, logo signs, package displays and other forms of advertising and promotion were recorded. Where possible, photographs of visible marketing displays were taken.

**RESULTS:**

Advertising of tobacco products was present in 2/3 of the nightclubs observed. The most commonly advertised product category were heated tobacco products. Tobacco brand representatives promoted and offered consumers free tobacco product samples. Multi-level marketing activities were observed, including tobacco branded bar accessories, prominent sale points and tobacco brand logo signs.

**CONCLUSIONS:**

The majority of nightclubs observed in Gdansk advertise and promote tobacco products. The ban on tobacco advertising and promotion is being violated in nightclubs, where the most commonly advertised products are heated tobacco products. The authorities should take steps to extend the bans to cover private promotion and enforce the law to protect high-risk individuals from smoking initiation and relapse.

## INTRODUCTION

According to the World Health Organization estimates, 8 million people die each year due to tobacco use^1^. Eurobarometer data from 2021 show that 54% of current and former smokers started smoking before the age of 18 years, with 38% initiating between the ages of 18 and 25 years^2^. According to the most recent data from Poland (2022), 23.8% of women and 28.9% of men aged 18–29 years smoke tobacco products^3^. This is of particular concern because people who start smoking at a young age are more likely to become daily smokers^4^.

Numerous measures, such as tobacco advertising bans and smoking restrictions, have been introduced worldwide to overcome the smoking epidemic. Although tobacco smoking is declining in developed countries, the use of heated tobacco products (HTP) is increasing at an alarming rate. This is because, in response to the restrictions, the tobacco industry introduces new solutions and products to remain on the market^5^. Between 2018 and 2020 alone, HTP sales in Europe increased from 934 million to 19.7 billion sticks, i.e. by 2000%^6^. In Poland, the sales of HTP sticks in 2018 amounted to 0.01 million units, but in 2020 reached 2.4 billion^7^.

HTP brands available on the Polish market among others are IQOS (Philip Morris International, PMI) and ‘glo’ (British American Tobacco, BAT). PMI advertises its products to consumers as a ‘harm-reduction’ alternative to cigarettes^8^. HTP devices are advertised as products of modern technology, stylish, with a simple design^9^.

Previous research shows that in order to target young adults, the tobacco industry is focusing its marketing strategy on social settings such as bars and nightclubs^10-12^. The industry exploits the association between drinking and a desire to smoke to advertise their products in these types of venues^13^. In Poland smoking at indoor premises is legal only if the venue provides a ventilated or filtered separate smoking room. Outdoor nightclubs or garden spaces belonging to indoor venues are not covered by this law.

According to the WHO Framework Convention on Tobacco Control (FCTC) guidelines, implementing comprehensive bans on tobacco advertising, promotion and sponsorships (TAPS) is an effective strategy of tobacco control policy^14^. The 1995 Polish Tobacco Control Act (TCA) forbids tobacco companies from advertising and promoting tobacco products^15^. The ban on advertising applies to: all tobacco products (incl. HTP), electronic cigarettes, refill containers, tobacco accessories or symbols related to them. The TCA defines advertising of tobacco products as ‘dissemination of messages, images of tobacco brands or symbols related to them, names and graphic symbols of tobacco product manufacturers which are used to popularize the tobacco product brands’. Promotion is described in particular as ‘public distribution of tobacco products, organizing tastings and other forms of encouragement to purchase or use tobacco products’. Since the 2016 amendment to the TCA, electronic cigarettes have also been covered by the ban and the definition of ‘novel tobacco products’ was added and clarified.

Bans introduced in Poland on tobacco advertising, promotion and sponsorship in media including broadcast, print and billboards are effective^16^. On the other hand, the compliance in hard-to-reach places such as nightclubs has not been researched in Poland. In a study conducted in Texas bars and nightclubs, where tobacco marketing is permitted in ‘adult only facilities’ 61% of respondents (college students) reported the presence of tobacco advertising, 23.2% observed the presence of free samples, and 25.8% noted the participation of representatives of tobacco brands^17^. This shows that the strategy of placing ads in nightclubs and bars is widespread, and despite the fact that each country has different regulations, this marketing continues to influence the young adults who visit these places. A study from Australia reported that PMI’s strategy for introducing HTP, which is currently banned in Australia, is to create a network of pubs, bars and clubs where these products can be sold after potential legalization^18^. The venues for this network have been chosen carefully, so as to reach mainly young adults and slowly build their bond with the brand.

There are not enough data about promotion and advertising of tobacco products in nightclubs in Poland. One study conducted in 2007–2009 in Warsaw showed that tobacco products were sold in 60% of nightclubs while advertisements were present in 50% of them^19^. More recent research is needed because since then, new tobacco products, such as HTP, have appeared on the Polish market, and they are aggressively advertised (2017). The aim of our study was to investigate the occurrence and characteristics of the advertising and promotion of tobacco products as it occurs after the launch of HTP, in nightclubs in Gdansk, a large city in northern Poland.

## METHODS

### Overview

We conducted a single-center observational study of nightclubs in the city of Gdansk area. This is the largest city in northern Poland in terms of number of inhabitants (486000 as of 2022)^20^. Gdansk is situated by the Baltic Sea which makes it a popular destination for tourists, especially during the summer. In 2021 Gdansk was visited by 3 million tourists^20^. The city offers various sightseeing attractions as well as hosting many concerts, festivals and other events during the high season, which also lead to increased tourist traffic. The study was conducted during the summer season between July and October 2022. Following a literature review, and due to the scarcity of similar studies in the literature, we based our study design mainly on the research conducted in the area of Boston, MA (USA) by Biener et al.^21^, which provided a detailed description of materials and methods used, and thus served as an inspiration for our project. Our observational data collection form and other aspects of the method were modified to be more actual and adjusted in line with our specific aims. Research methods were approved by the Independent Bioethics Committee for Scientific Research at the Medical University of Gdansk.

### Sampling frame

The research sample for this study was identified from all nightclub type venues in the city of Gdansk. For the purpose of the study we used the Cambridge dictionary definition of a nightclub: ‘a place that is open late into the night, where people can go to drink and dance and often see some type of entertainment’^22^. The venue had to meet the above criteria in order to be qualified for the study sample. We checked the character of each nightclub by searching online to establish their category and description of offered entertainment. The nightclubs were identified using: 1) the nightclubs and discos in Gdansk listed on the most popular local information portal in Gdansk (Trojmiasto.pl); 2) the listings in ‘Dance Clubs & Discos in Gdansk’ at TripAdvisor; and 3) the results of a Google keyword search on ‘nightclubs Gdansk’. After compiling the initial list, we also included two popular outdoor entertainment venues for young adults, especially popular in the summer. They are both open-air spaces, which offer food, alcohol, music from DJs and other attractions. While not strictly defined as ‘nightclubs’ by the criteria used in online listings, they met our inclusion criteria. Pubs were excluded because they do not provide dancing or entertainment. We also excluded venues that were classified as ‘adult entertainment venues’.

### Study material

A total of 38 venues met the ‘nightclub’ selection criteria and qualified for the study. Three nightclubs were removed from the list because they were closed for the summer season, leaving 35 in the final sample. We completed the observation in 30 out of the 35 nightclubs during the selected observational period (7 July to 1 October). The remaining 5 clubs were closed to the public for private events or concerts on the day selected for the visit.

### Data collection

The study was carried out from 9 p.m. to 1 a.m. on Thursdays, Fridays and Saturdays. Two observers independently collected data and recorded their observations in the same venue at the same time. They were called observers A and B (a 22-years-old female and a 23-years-old male). Observers were trained before participating in the study, and they were briefed with the aim and methods of the research. They were familiarized with the data collection form (Supplementary file) and the definitions of ‘advertising’ and ‘promotion’. We provided them with the information about what they should pay attention to in terms of tobacco marketing. At the end of the 2-h training sessions they completed a pilot visit (a venue thus visited was assessed again during the main part of the study), and their observations were reviewed and discussed. The observation period was adjusted to the size of the nightclub, and in each case was at least 30 minutes duration. During the observation, the observers checked the whole space and all rooms in the nightclub and completed the previously prepared form on their cell phones in real time. Where possible, observers took photographs of the advertisements, promotions and displays of tobacco products that were present. If representatives of tobacco brands were spotted in the club, the observers approached them to talk about the promotion they were offering. Observers were responsible for collecting any tobacco-related free samples and/or other promotional items (‘gadgets’) that were distributed. After leaving the club, the observers transferred the data from mobile phones to a paper version of the data collection form for archiving purposes.

### Observational data

The collected data included: nightclub type (indoor club, club with an outdoor zone, outdoor club); entertainments/attractions (DJ, photo booth, karaoke, live music); a designated smoking area (outdoor or indoor); entrance fee; and whether the age of the observers was checked on entry. The presence of tobacco sale points and types of tobacco products sold were also recorded as was the presence of advertising (tobacco packages/ devices display, logo signs, branded bar accessories). Observers also noted types and names of branded bar accessories (dustbins, ashtrays, deck chairs, tables, straw stands, bar mats) and active promotions including representatives of tobacco brands, distribution of free samples and free gadgets. Free tobacco samples included packs of tobacco products or a tobacco products tasting offered by tobacco brand representatives. ‘Gadget’ was understood as a branded promotional item from tobacco companies (lighters, matches, cigarette cases, etc.). Observers recorded the names of the tobacco brands available for sale at the club. At the end of the form there was a space for observers’ comments and reports from conversations with tobacco brand representatives. Data collected by observers A and B were discussed, compared and resolved in case of differences.

### Data analysis

The data from the observation forms were gathered in a Microsoft Excel spreadsheet database (part of the Office 365 package), which was also used to compute basic quantitative statistics. The photos were labelled and placed in a separate digital folder.

We also reviewed the qualitative comments made by the observers (answers to open-ended questions at the end of the form), photographs and quantitative data, and selected three nightclubs that stood out in terms of tobacco marketing to describe as case studies. These venues were characterized by the presence of different marketing activities, promotions and prominent points of sale.

## RESULTS

Table 1 presents general data about the nightclubs and tobacco marketing observed there. The majority of the nightclubs we visited were indoor or indoor with outdoor zones. Most venues allowed smoking outside the club, with indoor smoking observed only in 4 nightclubs. Half of the investigated nightclubs offered free entry. A third of the venues verified the age of our observers. The most common type of entertainment was a DJ. The next part of the table presents tobacco promotional and advertising activities recorded in the observed nightclubs. Sale points were present in 70% of the nightclubs. Advertising activities were recorded in the majority of the observed venues, while promotional activities took part in a small number of the venues at the time of the observation.

**Table 1:** General characteristics of nightclubs and observed tobacco marketing, Gdansk, Poland, 2022 (N=30)

*Characteristics*	*% (n)*
**Type of nightclub**	
Outdoor	16.7 (5)
Indoor	46.7 (14)
Indoor with an outdoor zone	36.7 (11)
**Entrance**	
Free	50.0 (15)
Fee paid	50.0 (15)
**Observers’ age verified**	
Yes	33.3 (10)
No	66.7 (20)
**Designated smoking area**	
Outside the club	70.0 (21)
All the area*	16.7 (5)
Smoking room inside	13.3 (4)
**Entertainment**	
DJ	76.7 (23)
Foodtruck	10.0 (3)
Karaoke	3.3 (1)
Photobooth	3.3 (1)
Other	6.7 (2)
**Sale**	
Tobacco products sale point	70.0 (21)
**Advertising**	
Tobacco brand logo display (signs)	66.7 (20)
Tobacco packages/devices display	63.3 (19)
Branded bar accessories	66.7 (20)
**Promotion**	
Tobacco brand representatives	10.0 (3)
Free tobacco product samples	10.0 (3)
Free gadgets	6.7 (2)

* Only for outdoor nightclubs.

Table 2 presents detailed data on advertising and promotions in a subsample of nightclubs (n=20) where such activities were observed. ‘Camel’ and ‘glo’ were the brands that appeared the most frequently on bar accessories. In total, 60 % of nightclubs’ bar accessories had a logo of an HTP brand and 45% had accessories with a cigarette logo. Only in one nightclub, two different brands of tobacco products were advertised concurrently. In other venues only one tobacco brand was advertised and promoted. Our observations showed that 75% of tobacco brand logo displays were located at the bar, where the alcohol was sold. Three nightclubs, all classified as student clubs, did not promote or advertise tobacco products.

**Table 2:** Tobacco product types and brands present in observed nightclubs, Gdansk, Poland, 2022 (N=20)

*Variable*	*% (n)*
**Type of tobacco products on branded bar accessories**	
HTP	60.0 (12)
Cigarettes	45.0 (9)
**Brand of tobacco product on branded bar accessories**	
Glo	35.0 (7)
IQOS	25.0 (5)
Camel	35.0 (7)
Pall Mall	10.0 (2)
**Bar accessories**	
Dustbins	65.0 (13)
Bar mats	45.0 (9)
Tables and deck chairs	35.0 (7)
Straw stands	35.0 (7)
**Brand of tobacco product on logo signs**	
Glo	30.0 (6)
IQOS	35.0 (7)
Camel	30.0 (6)
Pall Mall	10.0 (2)
**Tobacco brand logo sign location**	
Bar	75.0 (15)
Dance floor	25.0 (5)
Smoking room	10.0 (2)
Chillout zone	15.0 (3)

### Case study venue 1

Venue 1 was an outdoor club in a popular party location, with a large, 2-storey high HTP brand zone (Supplementary file Figures 1a and 1b). The zone had been designed in an elegant, minimalist style with atmosphere-creating lighting. There were tables and seating in the zone. There was also a sale point where both HTP and gadgets with the tobacco brand logo could be bought. Representatives of the tobacco brand offered free tastings of heated tobacco products.

### Case study venue 2

Venue 2 was another open-air nightclub. This venue featured a chillout zone with furniture and accessories marked with the tobacco brand logo, including deckchairs, tables, ashtrays, as well as illuminated tobacco brand logo signs (Supplementary file Figure 2a). In addition to the sale of HTP at the bar, there was a bus-shaped sale point (Supplementary file Figure 2b). The graffiti on the ‘bus’ makes a play on words in Polish: the phrase ‘Tuse podgrzewam’ means ‘Here I heat up’, where ‘Tuse’ is both a stylized colloquial rendition of a phrase meaning ‘Here I’ and a name of a well-known local graffiti artist. Representatives of the tobacco brand encouraged the purchase of the device and offered a free tasting of the HTP.

### Case study venue 3

Venue 3 had an artificial beach, complete with branded furniture and accessories: deckchairs, tables, ashtrays and neon signs (Supplementary file Figure 3a). Tobacco brand representatives encouraged observers to play a cellphone game, which consisted of finding a colored animal on the screen (Supplementary file Figure 3b). The tobacco representative asked about the age of the observer who wanted to play the game, but did not verify it. The game appeared on the screen after scanning the QR code. After winning in the game, the observer received a free sample pack of cigarettes as a reward. In addition to the prominent tobacco brand presence in the club described above, in two other clubs we observed displays of multimedia animation with the logo of the same brand.

## DISCUSSION

The study is, to our best knowledge, the first study on the marketing of tobacco products, including HTP, in nightclubs in Poland. So far, very little research has been carried out in Poland that involves actual observation of tobacco marketing occurring in public places. Most previous studies only covered points of sale (POS) advertising at typical retail outlets such as supermarkets or other grocery stores where multiple brands are usually available and did not include data collection from hard-to-reach places where tobacco concessions are granted to specific brands. The study clearly demonstrated four key findings: 1) advertising of tobacco products was present in the majority of nightclubs observed in Gdansk; 2) while HTP were the most frequently advertised product, traditional cigarette advertising was still present in the observed venues; 3) tobacco marketing in nightclubs consisted of high-intensity, highly visibility, several channels promotions and advertising; and 4) the TCA laws were violated.

According to Article 13 of the WHO FCTC, it is essential to implement comprehensive bans on all forms of tobacco promotion and advertising to eliminate possible loopholes in the law^15^. Furthermore, it is important to set out the law in a way that prevents tobacco companies from evolving their products and marketing strategies to exploit loopholes or create ‘workarounds’. Although Poland implemented the majority of the WHO FCTC Article 13 guidelines in the TCA, our results clearly indicate that these provisions are not adequately enforced and that non-compliance is common in hard-to-reach places, e.g. nightclub settings.

Studies conducted in Poland which investigated POS marketing of tobacco products support the observation that despite the ban, the law is not obeyed. A study from the city of Lodz, Poland, before 2014 and after 2019, the introduction of the electronic cigarettes advertising ban showed no significant differences in the presence of advertisements^16^. Another observational research on POS marketing reported non-compliance with the law in 79.5% of POS^23^.

Enforcing the law through physical observation of each venue requires more effort and resources allocated to the task. The problem of tobacco marketing in nightclubs might seem to be less important or even non-existing as it is not as prominent as, for example, television advertising. It is, however, crucial for responsible governmental agencies to effectively control and enforce the bans in order to stop the tobacco companies’ aggressive marketing tactics.

In addition to effective enforcement of existing bans, another issue is that the Polish TCA defines ‘tobacco products promotion’ as ‘public distribution’. This means that promotion which has a private character is not covered by the ban. This leads to the tobacco companies offering ‘private promotion’ to consumers and thus evading the law. During our study, we observed tobacco brand representatives approaching observers ‘privately’ in order to offer them tobacco products. The 1995 TCA should be amended with a ban on all forms of tobacco promotion, both indirect and direct, public and private, in order to eliminate such practices^24^.

Despite bans and regulations, tobacco companies still engage in aggressive marketing. In particular, the appearance of HTP on the market has created a major threat to public health. The tobacco industry claims that HTP reduce smokers’ health risk^25^. Our study results show that marketing of these products targets young adults, not older heavy smokers, which is in marked contrast to the tobacco industry’s narrative. A study from 2021 showed that the use of HTP in Poland is not replacing traditional cigarettes, in contrast with, for example, Japan, where such a trend has been noticed^26^.

### Limitations

The limitations of the study include the reduced nightclubs’ sample due to the 5 venues which were closed or held a private event on the day of the observation. It is possible that this did not have a major impact on the result of the study because these clubs were not markedly different from the others, so similar results would have been expected there. The results are also limited to presenting the problem only in the Gdansk city area. Tobacco companies may be more willing to aggressively advertise their products in cities where they have a large target group (young adults). This study was conducted during the high season, and only represents data from this period. Some outdoor nightclubs are only open during the summer and other nightclubs open up additional outdoor spaces, which can result in a higher intensity of tobacco marketing. Therefore, the results should be interpreted with caution when generalizing to smaller or less popular cities.

## CONCLUSIONS

This study sheds light on the marketing practices of the tobacco industry in nightclubs in Poland with the example of the city of Gdansk. Promotion and advertising of tobacco products was found in the majority of nightclubs observed. Marketing activities stand out with high intensity and exposure. HTP are the most widely advertised tobacco products. Large and prominent tobacco brand points of sale are present, as well as numerous accessories signed with the brand logo. The law prohibiting tobacco marketing is being violated in Gdansk and the enforcement of this law is needed.

## Data Availability

The data supporting this research are available from the authors on reasonable request.
