# Supplementary material for: Heated tobacco products and cigarette marketing in nightclubs in Gdansk, Poland: A mixed-methods analysis
Source: Tob Prev Cessat. 2024 Jan 5;10:10.18332/tpc/174573. doi: 10.18332/tpc/174573 (PMC10768186; doi:10.18332/tpc/174573)
Supplement: Supplementary file 1 [file TPC-10-01-s1.pdf]

RESEARCH FORM

Observation of advertising and promotion of tobacco products in nightclubs   observer A B

**Name of venue**		**Date:**	
**Adress**		**Time of entrance:**	
**Type of the venue**		☐ outdoor ☐ indoor ☐ indoor with outdoor zone	

**1.General information**	**Details**		**Comments**
Age verification	☐ yes ☐ no		
Designated smoking area	☐ outside the club ☐ all over the area (for outdoor clubs) ☐ smoking room inside		
Enteirtainment	☐ karaoke ☐ photobooth ☐ DJ ☐ foodtrucks ☐ other ☐ none		
Entrance fee	☐ free ☐ paid, amount:		
**2.Advertising**		**Details**	**Comments**
Branded logo signs	☐ yes ☐ no	**Location:** ☐ at the entrance to the club ☐ at the bar ☐ at dancefloor ☐ in smoking area ☐ other	
Bar accessories	☐ yes ☐ no	**Type:** ☐ bar pads ☐ dustbins ☐ deck cheirs ☐ ashtrays ☐ tables ☐ other	
Tobacco packages/devices display	☐ yes ☐ no		
Brand of above advertising	☐ glo ☐ iqos ☐ logic ☐ Pall Mal ☐ Camel ☐ other		
**3. Promotion**		**Details**	**Comments**
Free tobacco products samples	☐ yes ☐ no	☐ free tobacco tasting ☐ free sample (pack of tobacco products) ☐ other	
Free gadgets	☐ yes ☐ no	☐ matches ☐ lighters ☐ other	
Brand of above promotion	☐ glo ☐ iqos ☐ logic ☐ Pall Mall ☐ Camel ☐ other		
**4. Sale**		**Details**	
Tobacco products sale point	☐ yes ☐ no	Type: ☐ separate point ☐ at the bar ☐ other	
Tobacco products types, available for sale	☐ cigarettes ☐ e-cigarettes ☐ liquids ☐ HTP ☐ other		
Brand available for sale	☐ glo ☐ iqos ☐ logic ☐ Pall Mall ☐ Camel ☐ other		
**5.Tobacco brand representatives**	☐ yes ☐ no	**Details**	**Comments**
Encouraging to fill the survey	☐ yes ☐ no	**What purpose?** ☐ receiving free samples ☐ receiving free gadgets ☐ other<br>Questionnaire: ☐ age ☐ smoking status ☐ type of products used ☐ other	
Brand	☐ glo ☐ iqos ☐ logic ☐ Pall Mall ☐ Camel ☐ other		

Comments	

Figure 1a and 1b

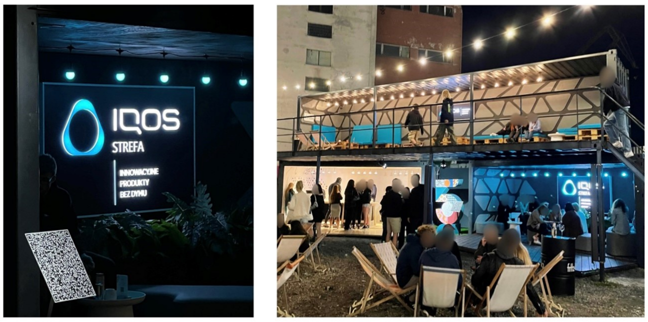

Figure 2a and 2b

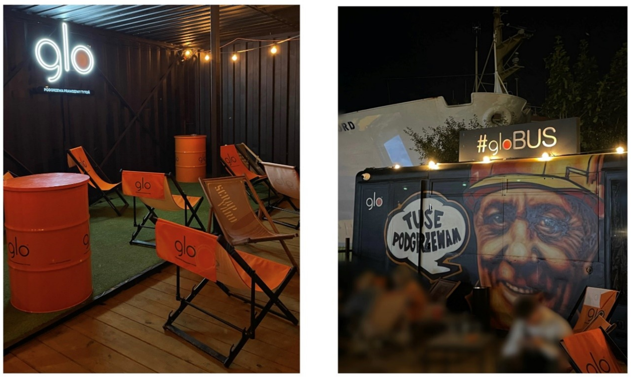

Figure 3a and 3b

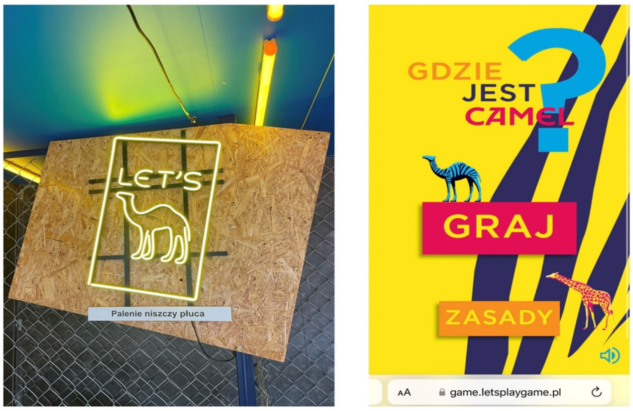

© 2023 Nowika J. et al.
